# Cellular homeostatic tension and force transmission measured in human engineered tendon

**DOI:** 10.1016/j.jbiomech.2018.07.032

**Published:** 2018-09-10

**Authors:** Antonis Giannopoulos, Rene B. Svensson, Katja M. Heinemeier, Peter Schjerling, Karl E. Kadler, David F. Holmes, Michael Kjaer, S. Peter Magnusson

**Affiliations:** a Institute of Sports Medicine Copenhagen, Department of Orthopedic Surgery, Bispebjerg-Frederiksberg Hospital and Center for Healthy Aging, Faculty of Health Sciences, University of Copenhagen, Denmark; b Department of Physical and Occupational Therapy, Bispebjerg-Frederiksberg Hospital, Copenhagen, Denmark; c Wellcome Trust Centre for Cell-Matrix Research, Faculty of Biology, Medicine and Health, Manchester Academic Health Science Centre, University of Manchester, Manchester M13 9PT, UK

**Keywords:** Cell-matrix interaction, Fibroblast, Force monitor, Mechanics, Tissue engineering

## Abstract

Tendons transmit contractile muscular force to bone to produce movement, and it is believed cells can generate endogenous forces on the extracellular matrix to maintain tissue homeostasis. However, little is known about the direct mechanical measurement of cell-matrix interaction in cell-generated human tendon constructs. In this study we examined if cell-generated force could be detected and quantified in engineered human tendon constructs, and if glycosaminoglycans (GAGs) contribute to tendon force transmission. Following de-tensioning of the tendon constructs it was possible to quantify an endogenous re-tensioning. Further, it was demonstrated that the endogenous re-tensioning response was markedly blunted after interference with the cytoskeleton (inhibiting non-muscle myosin-dependent cell contraction by blebbistatin), which confirmed that re-tensioning was cell generated. When the constructs were elongated and held at a constant length a stress relaxation response was quantified, and removing 27% of the GAG content of tendon did not alter the relaxation behavior, which indicates that GAGs do not play a meaningful role in force transmission within this system.

## 1 Introduction

The chief function of tendon is to transmit contractile muscular force to bone to produce movement. It has been shown that placing sizeable repetitive loads on the tendon may influence numerous cell responses (Spiesz et al., 2015), tissue composition (Langberg et al., 1999) and mechanical properties of the tendon (Hansen et al., 2003), which indicates that tendon tissue is mechanoresponsive although the precise pathway is unknown (Harris et al., 1980, Wang et al., 2012). This conversion of a mechanical stimulus into an electrochemical action and intracellular biochemical response demonstrate that tendons are capable of mechanotransduction. While the tendon can impart forces on the cell, it is also possible for cell to generate endogenous forces on the extracellular matrix (ECM) (Eastwood et al., 1994, Kolodney and Wysolmerski, 1992), which allows for a fine-tuned dynamic interaction between the cell and the ECM to maintain tissue homeostasis (Freedman et al., 2015, Joshi et al., 1985).

The ability for cells to exert forces on the ECM has previously typically been quantified using a polymeric collagen lattice to show that cells can control homeostatic tension when measured over hours to days (Delvoye et al., 1991, Eastwood et al., 1994). Sponge gels with defined properties have also been used as scaffolds to evaluate cell responses (Brown et al., 1998, Delvoye et al., 1991, Kolodney and Wysolmerski, 1992). However, cell-generated scaffolds comprise a mixture of ECM components that more likely resemble that of the in vivo situation compared to the aforementioned models. Such cell-derived tendon construct have therefore been developed using both animal (Kapacee et al., 2008) and human cells (Bayer et al., 2010), with similar composition (Kapacee et al., 2008) and mechanical properties (Herchenhan et al., 2013) to embryonic tendon tissue (Kalson et al., 2010). Tension appears critical for the formation and development of these constructs, which underscores the importance of mechanotransduction (Bayer et al., 2014, Kapacee et al., 2008). However, direct mechanical measurement of cell-matrix interaction in cell-generated human tendon constructs has never been reported before.

The principle force transmitting structure of the ECM in mature tendon tissue is the fibril (Cribb and Scott, 1995, Parry et al., 1978). It has been suggested that force is transferred between adjacent fibrils via proteoglycans and their associated glycosaminoglycan (GAG) chains, including chondroitin- and dermatan-sulfate (Ryan et al., 2015, Scott and Thomlinson, 1998). However, removing this complex in tendon (Svensson et al., 2011) and ligament (Provenzano and Vanderby, 2006) does not appreciably affect the mechanical properties of the tissue. Moreover, it was recently shown that fibrils appear to be continuous in mature tendon tissue, suggesting that the importance of lateral force transmission between fibrils may be negligible (Svensson et al., 2017). However, in the early stages of developing tendon tissue the fibrils are discontinuous (Birk et al., 1995), and the relative amount of non fibrillar matrix is larger. Therefore it is possible that a mechanism for lateral force transmission is necessary, but this has never been investigated. Hence, the purpose of this study was two-fold; (1) to examine if cell generated force could be detected and quantified in engineered human tendon constructs, and (2) to assess if GAGs contribute to transmission of force in this human cell generated tendon tissue.

## 2 Materials and methods

### 2.1 Tendon construct preparation

Supplementary data associated with this article can be found, in the online version, at https://doi.org/10.1016/j.jbiomech.2018.07.032.

Cells were obtained as previously described (Bayer et al., 2010) (see supplement for details). In brief, tendon fibroblasts were isolated from semitendinosus and gracilis tendon from patients that underwent reconstructive anterior cruciate ligament (ACL) surgery. All the cell lines from different donors were obtained from the same source. Informed consent was obtained from all tissue donors in accordance with ethical approval [H-3-2010-070]. Cells were isolated using collagenase type II and seeded into culture flasks (DMEM/F12, 10% FPB). Cells between passages 2 and 6 were used for experiments.

**Supplementary material:** 

Tendon constructs from human tendon fibroblasts were made as previously described (Bayer et al., 2010) (see supplement for details). Briefly, each well of a six well plate was coated with Sylgard (DoW Chemicals). Two loop shaped silk sutures were pinned 10 mm apart to the coated plates and sterilized in 70% ethanol. Fibroblasts were suspended in a mix of fibrinogen, aprotinin and thrombin (all from Sigma Aldrich) to a final concentration of 0.2 million cells per well. The 3D gels were incubated in construct medium (DMEM/F12, 10% FBS, 0.2 mM L-ascorbic acid 2-phosphate, 0.05 mM L-proline), which was replaced every other day. Approximately 2 weeks after seeding the constructs were fully formed (the matrix contracted to a 10 mm long narrow linear structure between the sutures).

### 2.2 Mechanical evaluation

**Fig. 1:**
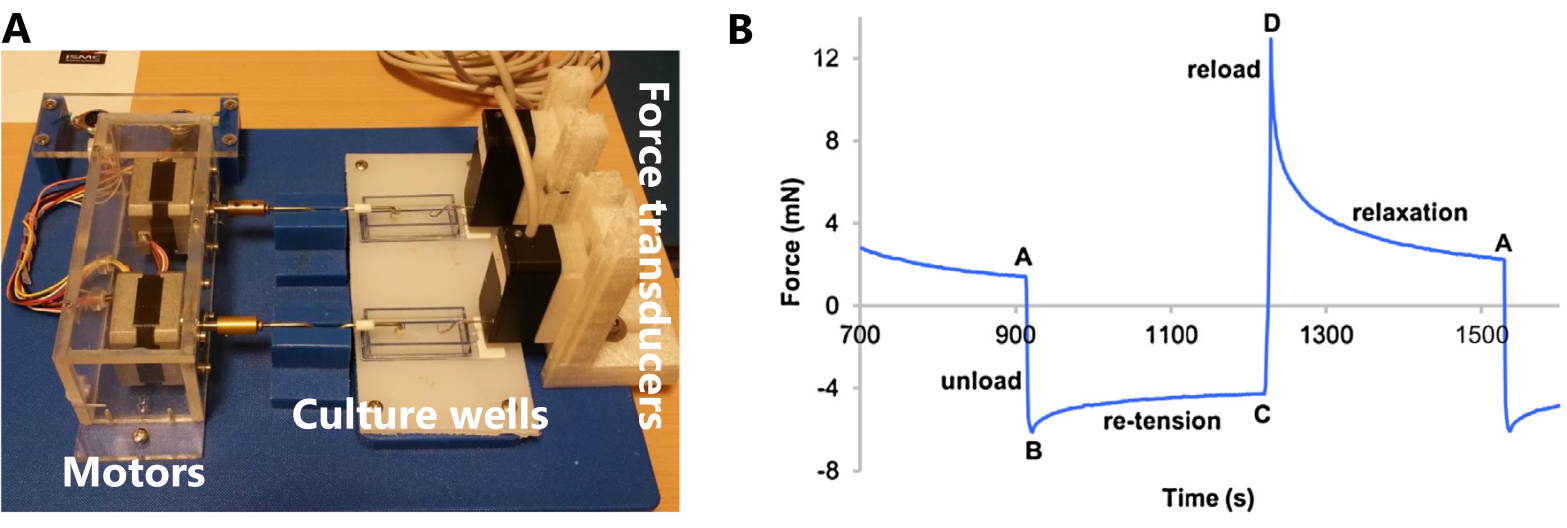
Mechanical test system. (A) The force monitor system which consists of two stepper motors, culture wells and force transducers. (B) Example of force vs. time data for one cycle measurement. At point ‘A’ the construct is unloaded to point ‘B’ where the force is allowed to re-tension for 300 s. At point ‘C’ the construct is stretched back to its initial position (point ‘D’) with the length remaining constant for 300 s, relaxing back to point ‘A’ where another cycle starts.

A custom made system was used to measure forces in cell derived human tendon constructs. Briefly, the system consisted of force transducers (402A, Aurora Scientific, CA), stepper motors with a motor controller (Astrosyn, Y129-5, PC-control ltd., UK), culture wells and a PC data collection system (Microlink 751, Biodata ltd., UK). Strain was applied by the stepper motors via a threaded rod with a step resolution of 2.25 µm. Deformation was applied at a rate of 56 µm/s and force data sampled at 1 Hz. Constructs were attached by their silk suture loops to the motor and force transducer via stainless steel hooks (Fig. 1A).

### 2.3 Mechanical testing protocol

Mechanical tests were performed in an incubator (37 °C and 5% CO_2_). The Sylgaard coating underneath the construct was cut into a strip and transferred together with the pinned construct to the force monitoring system to avoid altering the original length and tension. Thereafter, the constructs were relaxed by 0.225 mm to confirm the presence of tension. If tension was present the length was returned to the original position. If there was no tension the constructs were considered to have become slightly slack during transfer and were stretched in 0.225 mm steps up to 0.675 mm to re-establish tension. This position was defined as the baseline length and subsequently the system was allowed to stabilize for 1 h. The tendon constructs were subjected to a protocol that consisted of three cycles, with each cycle consisting of 0.675 mm of unloading (reducing length), 300 s of rest period followed by 0.675 mm of reloading (returning to the initial length) and another 300 s of rest (see Fig. 1B).

### 2.4 Construct treatment

Tendon constructs were tested at either 3, 4 or 5 weeks after seeding (based on 5 cell lines). The tendon constructs underwent three cyclic stretches in normal medium (DMEM). Immediately after, the normal medium was replaced with treatment media (DMEM plus reagent) followed by an incubation period of 30 min and three subsequent cycles. Blebbistatin (B0560, SIGMA) (17 µM) was used for inhibiting non-muscle myosin-dependent cell contraction (n = 24: week 3, n = 8, week 4, n = 7, week 5, n = 9), and chondroitinase ABC (C3667, SIGMA) (0.07U/ml) was used to digest glycosaminoglycans (n = 23: week 3, n = 8, week 4, n = 7, week 5, n = 8). Control samples (n = 10 from 2 cell lines: week 3, n = 4, week 5, n = 6) that had normal medium replaced with fresh normal medium were also mechanically tested in the same manner to control for the effect of time (untreated controls).

### 2.5 Glycosaminoglycan determination

Sulfated GAG content was determined in the mechanically tested constructs using a 1,9-dimethylmethylene blue (DMMB) assay slightly modified from (Hoemann, 2004) and was expressed as µg per construct (see details in supplement).

### 2.6 Statistics and data reduction

The force values were determined at four different points in each cycle (see Fig. 1B): A) at the end of relaxation (300 s). B) Immediately following unloading. C) At the end of re-tension (300 s). D) Immediately following reloading. Average values of the 3 cycles were used for each sample before and after treatment. Re-tension was calculated as: (C − B)/(A − B), which corresponds to the relative amount of re-tension. Stress relaxation was calculated as: (D − A)/(D − C), which also corresponds to the relative amount of relaxation. Re-tension and relaxation are expressed as a percentage and the treatment effect is the absolute difference between the pre and post percentage values.

The effect of treatment and construct maturity on mechanical behavior was examined with 2-way ANOVA’s with post hoc Sidak’s multiple comparison tests (GraphPad Software, La Jolla California USA). Unpaired t-tests were used to compare GAG content and change in re-tension between chondroitinase and blebbistatin treated constructs and to compare the baseline mechanics (before treatment) between week 3 and 5. The primary comparison was between blebbistatin and chondroitinase treated samples but as an additional control, unpaired t-tests were also made against the untreated controls. Results are reported as mean ± SE.

## 3 Results

**Fig. 2:**
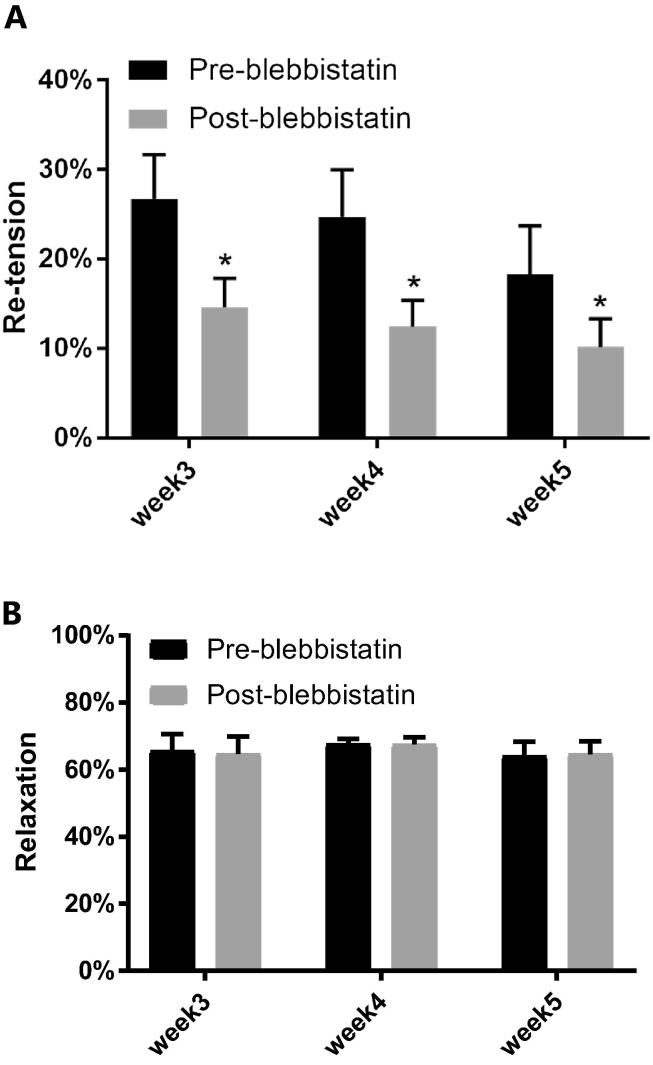
Blebbistatin treatment for cell contraction inhibition. (A) The re-tension dropped significantly after blebbistatin treatment (p < 0.005). B) The relaxation phase was unaffected by cell contraction inhibition. (n = 24 from 5 cell lines: week 3, n = 8, week 4, n = 7, week 5, n = 9).

**Fig. 3:**
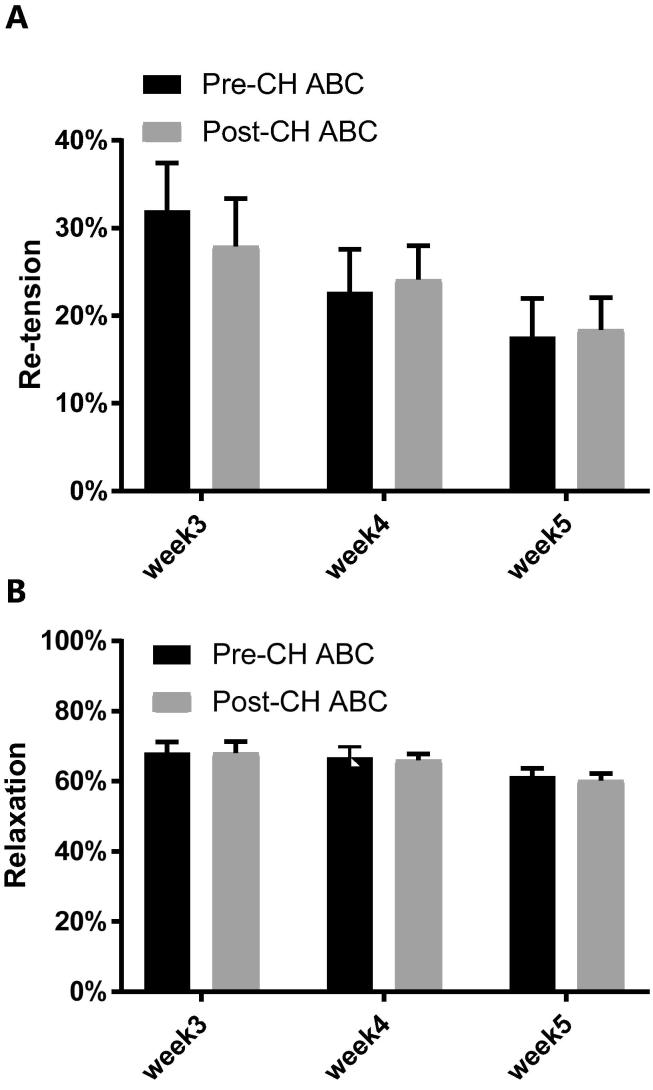
Chondroitinase ABC (CH ABC) treatment for glycosaminoglycan digestion. (A) The re-tension did not change after CH ABC treatment. (B) The relaxation also remain unaffected after the treatment. (n = 23 from 5 cell lines: week 3, n = 8, week 4, n = 7, week 5, n = 8).

In untreated controls re-tension decreased over time (−3.5 ± 1.2%, n = 10, p < 0.05) but relaxation was unaffected (−0.8 ± 0.5%, n = 10, p = 0.14) indicating little effect of time. Blebbistatin treatment significantly reduced re-tension compared to pre-treatment (Fig. 2A, p < 0.005, main effect) but had no effect on relaxation (Fig. 2B, p = 0.97, main effect). Blebbistatin treatment did not affect GAG content (7.17 ± 0.35 µg, n = 29) compared to the untreated controls (6.84 ± 0.63 µg, n = 7). Chondroitinase treatment reduced the total GAG content of the tendon constructs (5.24 ± 0.22 µg, n = 27) by 27% compared to blebbistatin treated constructs (p < 0.0001). Chondroitinase did not affect re-tension (Fig. 3A, p = 0.87, main effect) or relaxation (p = 0.74, main effect) of the constructs (Fig. 3).

The change in re-tension with blebbistatin (-10.6 ± 1.6%, n = 24) was significantly different from the change with chondroitinase treatment (−0.8 ± 1.5%, n = 23, p < 0.0001) and from the change in untreated controls (p < 0.05). While the effect of treatment did not differ between weeks there was a baseline reduction in re-tension from week 3 (29.4 ± 3.6%, n = 16) to 5 (18.0 ± 3.4%, n = 17, p = 0.029). There was no significant baseline difference in relaxation from week 3 (66.9 ± 2.8%, n = 16) to 5 (62.8 ± 2.4%, n = 17, p = 0.165).

## 4 Discussion

In the present study we sought to examine if cell-generated force could be detected and quantified in engineered human tendon constructs. When the constructs were unloaded an endogenous force was generated that could be quantified (see Fig. 1A), and following inhibition of cell contractility by blebbistatin (an inhibitor of non-muscle myosin) (Cai et al., 2006, Even-Ram et al., 2007, Kalson et al., 2013), the re-tension was dramatically reduced, which indicates that the endogenous force is cell-generated. Complete loss of re-tension did not occur, which may relate to the short treatment time.

The fact that the cells have the ability to generate internal tissue tension and thereby maintain homeostasis is well known and has been studied in different models. However, to the best of our knowledge, this is the first report of cell-generated tissue tension in a 3D human tendon scaffold. The advantage of this model is that the cells produce and organize their own collagen matrix (Herchenhan et al., 2013, Kalson et al., 2010).

The magnitude of re-tensioning was less in week 5 compared to week 3 constructs. This could be the result of a lower cell number, but an assessment of nuclei area fraction did not reveal a difference between the two weeks (see supplement). However, others have shown that cell number declines over time in similar constructs (Delvoye et al., 1991, Kalson et al., 2010). On the other hand, it has been shown that the stiffness of the construct increases dramatically from week 2 to 5 (Herchenhan et al., 2013), and it is possible that the increased collagen stiffness itself reduces the magnitude of the cell contraction (Karamichos et al., 2007). The relative contribution of a potential decline in cell number or augmented construct properties cannot be ascertained in the present study.

While the unloading allowed for an evaluation of the cell-generated response, the relaxation phase represents the response of the extracellular component of the matrix. As mentioned earlier, GAGs could influence force transmission in the immature constructs. However, in the present study we show that a 27% reduction of GAG content did not impact the relaxation phase of the 3–5 week constructs, which implies that this is not a critical force transmission pathway in these tissues.

In conclusion, these data show the presence of cell-generated tension within the human tendon constructs. This observation was supported by a 38% decline in cell re-tension after introducing a cell contraction inhibitor. In addition, a 27% reduction in GAG content did not seem to affect force transmission in this system. The ability of our system to apply, detect and quantify the generated forces in real time provides new insight to the field of tendon biomechanics. The force monitor is a useful tool to investigate tissue development and regeneration by evaluating cell-matrix interactions.

## 5 Conflict of interest statement

All authors declare no conflicting interests.

## References

[b0005] Bayer M.L., Schjerling P., Herchenhan A., Zeltz C., Heinemeier K.M., Christensen L., Krogsgaard M., Gullberg D., Kjaer M. (2014). Release of tensile strain on engineered human tendon tissue disturbs cell adhesions, changes matrix architecture, and induces an inflammatory phenotype. PLoS One.

[b0010] Bayer M.L., Yeung C.Y., Kadler K.E., Qvortrup K., Baar K., Svensson R.B., Magnusson S.P., Krogsgaard M., Koch M., Kjaer M. (2010). The initiation of embryonic-like collagen fibrillogenesis by adult human tendon fibroblasts when cultured under tension. Biomaterials.

[b0015] Birk D.E., Nurminskaya M.V., Zycband E.I. (1995). Collagen fibrillogenesis in situ: fibril segments undergo post-depositional modifications resulting in linear and lateral growth during matrix development. Dev. Dyn..

[b0020] Brown R.A., Prajapati R., McGrouther D.A., Yannas I.V., Eastwood M. (1998). Tensional homeostasis in dermal fibroblasts: mechanical responses to mechanical loading in three-dimensional substrates. J. Cell Physiol..

[b0025] Cai Y., Biais N., Giannone G., Tanase M., Jiang G., Hofman J.M., Wiggins C.H., Silberzan P., Buguin A., Ladoux B., Sheetz M.P. (2006). Nonmuscle myosin IIA-dependent force inhibits cell spreading and drives F-actin flow. Biophys. J..

[b0030] Cribb A.M., Scott J.E. (1995). Tendon response to tensile stress: an ultrastructural investigation of collagen:proteoglycan interactions in stressed tendon. J. Anat..

[b0035] Delvoye P., Wiliquet P., Leveque J.L., Nusgens B.V., Lapiere C.M. (1991). Measurement of mechanical forces generated by skin fibroblasts embedded in a three-dimensional collagen gel. J. Invest. Dermatol..

[b0040] Eastwood M., McGrouther D.A., Brown R.A. (1994). A culture force monitor for measurement of contraction forces generated in human dermal fibroblast cultures: evidence for cell-matrix mechanical signalling. Biochimica et Biophysica Acta (BBA) – General Sub..

[b0045] Even-Ram S., Doyle A.D., Conti M.A., Matsumoto K., Adelstein R.S., Yamada K.M. (2007). Myosin IIA regulates cell motility and actomyosin-microtubule crosstalk. Nat. Cell Biol..

[b0050] Freedman B.R., Bade N.D., Riggin C.N., Zhang S., Haines P.G., Ong K.L., Janmey P.A. (2015). The (dys)functional extracellular matrix. Biochimica et Biophysica Acta (BBA) – Mol. Cell Res..

[b0055] Hansen P., Aagaard P., Kjaer M., Larsson B., Magnusson S.P. (2003). Effect of habitual running on human Achilles tendon load-deformation properties and cross-sectional area. J. Appl. Physiol..

[b0060] Harris A.K., Wild P., Stopak D. (1980). Silicone rubber substrata: a new wrinkle in the study of cell locomotion. Science.

[b0065] Herchenhan A., Bayer M.L., Svensson R.B., Magnusson S.P., Kjaer M. (2013). In vitro tendon tissue development from human fibroblasts demonstrates collagen fibril diameter growth associated with a rise in mechanical strength. Dev. Dyn..

[b0070] Hoemann C.D. (2004). Molecular and biochemical assays of cartilage components. Cartilage and Osteoarthritis. Methods Mol. Med..

[b0075] Joshi H.C., Chu D., Buxbaum R.E., Heidemann S.R. (1985). Tension and compression in the cytoskeleton of PC 12 neurites. J. Cell Biol..

[b0080] Kalson N.S., Holmes D.F., Kapacee Z., Otermin I., Lu Y., Ennos R.A., Canty-Laird E.G., Kadler K.E. (2010). An experimental model for studying the biomechanics of embryonic tendon: Evidence that the development of mechanical properties depends on the actinomyosin machinery. Matrix Biol..

[b0085] Kalson N.S., Starborg T., Lu Y., Mironov A., Humphries S.M., Holmes D.F., Kadler K.E. (2013). Nonmuscle myosin II powered transport of newly formed collagen fibrils at the plasma membrane. PNAS.

[b0090] Kapacee Z., Richardson S.H., Lu Y., Starborg T., Holmes D.F., Baar K., Kadler K.E. (2008). Tension is required for fibripositor formation. Matrix Biol..

[b0095] Karamichos D., Brown R.A., Mudera V. (2007). Collagen stiffness regulates cellular contraction and matrix remodeling gene expression. J. Biomed. Mater. Res..

[b0100] Kolodney M.S., Wysolmerski R.B. (1992). Isometric contraction by fibroblasts and endothelial cells in tissue culture: a quantitative study. J. Cell Biol..

[b0105] Langberg H., Skovgaard D., Petersen L.J., Bulow J., Kjaer M. (1999). Type I collagen synthesis and degradation in peritendinous tissue after exercise determined by microdialysis in humans. J. Physiol..

[b0110] Parry D.A., Barnes G.R., Craig A.S. (1978). A comparison of the size distribution of collagen fibrils in connective tissues as a function of age and a possible relation between fibril size distribution and mechanical properties. Proc. Royal Soc. B: Biol. Sci..

[b0115] Provenzano P.P., Vanderby R. (2006). Collagen fibril morphology and organization: implications for force transmission in ligament and tendon. Matrix Biol..

[b0120] Ryan C.N., Sorushanova A., Lomas A.J., Mullen A.M., Pandit A., Zeugolis D.I. (2015). Glycosaminoglycans in tendon physiology, pathophysiology, and therapy. Bioconjugate Chem..

[b0125] Scott J.E., Thomlinson A.M. (1998). The structure of interfibrillar proteoglycan bridges (shape modules') in extracellular matrix of fibrous connective tissues and their stability in various chemical environments. J. Anat..

[b0130] Spiesz E.M., Thorpe C.T., Chaudhry S., Riley G.P., Birch H.L., Clegg P.D., Screen H.R. (2015). Tendon extracellular matrix damage, degradation and inflammation in response to in vitro overload exercise. J. Orthop. Res..

[b0135] Svensson R.B., Hassenkam T., Hansen P., Kjaer M., Magnusson S.P. (2011). Tensile force transmission in human patellar tendon fascicles is not mediated by glycosaminoglycans. Connect. Tissue Res..

[b0140] Svensson R.B., Herchenhan A., Starborg T., Larsen M., Kadler K.E., Qvortrup K., Magnusson S.P. (2017). Evidence of structurally continuous collagen fibrils in tendons. Acta Biomater.

[b0145] Wang J.H., Guo Q., Li B. (2012). Tendon biomechanics and mechanobiology–a minireview of basic concepts and recent advancements. J. Hand Ther..

