# Supplementary material for: Cellular homeostatic tension and force transmission measured in human engineered tendon
Source: J Biomech. 2018 Sep 10;78:161–5. doi: 10.1016/j.jbiomech.2018.07.032 (PMC6135935; doi:10.1016/j.jbiomech.2018.07.032)
Supplement: Supplementary file 1 [file mmc1.docx]

# Materials and Methods

## Tendon construct preparation

Tendon fibroblasts were isolated from human semitendinosus and gracilis tendon from patients that underwent reconstructive anterior cruciate ligament (ACL) surgery. Informed consent was obtained from all tissue donors in accordance with ethical approval [H-3-2010-070]. Under aseptic conditions the tissue was minced into pieces of ~ 2 mm^3^ and digested overnight in DMEM/F12 (Gibco, Invitrogen) supplemented with 0.1% collagenase type II (Worthington) and 20% fetal bovine serum (FBS) (Gibco, Invitrogen). Following repeated washes in culture medium (DMEM/F12, 10% FBS) the cells were seeded into flasks and cultured until the next passage. Cells between passages 2 and 6 were used for experiments.

Tendon constructs from human tendon fibroblasts were made as previously described (Bayer et al., 2010). Briefly, each well of a six well plate was coated with Sylgard (DoW Chemicals) and incubated at 55 °C for 48 h. Two loop shaped silk sutures were pinned to the coated plates with minutien insect pins (0.1 mm diameter) (Fine Science Tools GmbH), 1 cm apart. Plates were sterilized in 70 % ethanol for 45 min. Human fibroblasts were suspended in a mix of human fibrinogen (4 mg/ml)(F3879), 1U thrombin (T6884) and bovine aprotinin (A3428)(10 µg/ml) (all from Sigma Aldrich) to a final concentration of 0.2 million cells per 815 µL and rapidly spread over the complete surface of the wells. The plate was left for 30 min at 37 °C and then 3 ml of construct medium (DMEM/F12 plus 10% FBS supplemented with 0.2 mM L-ascorbic acid 2-phosphate and 0.05 mM L-proline) was added to each well. Construct medium was replaced every other day. Constructs were considered to be fully formed when the matrix had contracted to a narrow linear structure between the sutures (2 weeks after seeding).

- 1. Glycosaminoglycan determination

Sulfated GAG content was determined in the mechanically tested constructs using a 1,9 dimethylmethylene blue (DMMB) assay slightly modified from (Hoemann, 2004) and was expressed as µg per construct. Standards of chondroitin sulfate (C3788, SIGMA) in water were used. Constructs (chondroitinase and blebbistatin treated) and 10 µL of standards were digested in 100 µL of papain solution (0.125 mg/mL papain (P3125, SIGMA), 10 mM L-cysteine, 100 mM sodium phosphate, 10 mM EDTA, pH 6.5 at 60 °C for 2 hours with vortexing every 30 min. Triplicates of 10 µL digested sample were transferred into an untreated 96-well plate and 190 µL of DMMB reagent (16 µg/mL DMMB (341088, SIGMA), 40 mM glycine, 40 mM sodium chloride, 9.5 mM HCl, pH 3.0) was added to each well. The absorbance at 540 nm minus 590 nm was used for quantification. Blebbistatin treated constructs were considered to be controls.

# Microscopy

## Cell quantification

Tendon constructs (n=4) were embedded (TissueTek, Sakura Finetek) and snap frozen in isopentane while pinned, at week 3 and 5 after formation. Sections (20 µm) were cut and stained with DAPI to visualize the nuclei, and three images (425 by 320 µm) per section were taken (one for each end and one of the middle part). Nuclei appeared fragmented (Fig.S1) prohibiting consistent counting, so instead the area fraction of nuclei was determined using color thresholding in FIJI (Schindelin et al., 2012) as a measure of cell density. Area of nuclei fraction in weeks 3 and 5 was evaluated with an unpaired t-test.

There was no significant difference in the area fraction of nuclei from week 3 (38 ± 3%) to week 5 (34 ± 4%, p=0.088).

## Cytoskeletal structure

Fluorescence microscopy images were obtained using laser confocal microscope (LSM 700, Axio Imager 2, Zeiss). A control and a blebbistatin treated construct were fixed while pinned with 10 % formalin for 25 min. Subsequently they were stained with Hoechst (nuclei) and phalloidin (F-actin) to visualize the cytoskeleton.

For blebbistatin treatment confocal microscope images showed a disruption in actin cytoskeleton with the presence of aggregates at the treated (Fig.S2.A) but not at the untreated controls (Fig.S2.B).

# Supplementary Figures


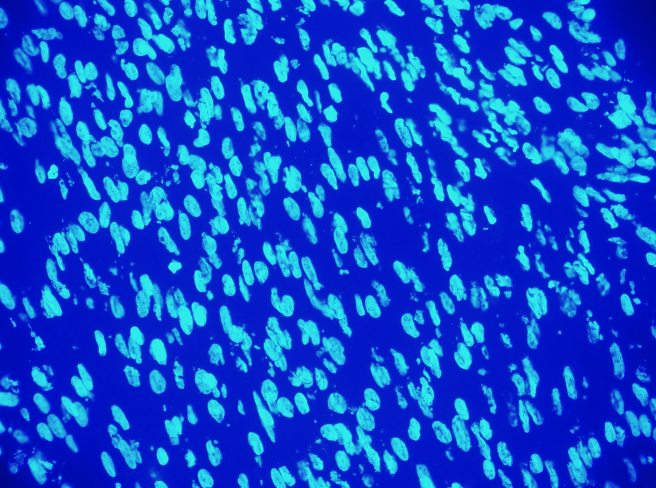


Fig.S1. Representative fluorescence image of human tendon construct stained with nuclear counterstain (DAPI). Red outlined section indicates the area analyzed for nuclei area fraction to exclude the edges of the construct.


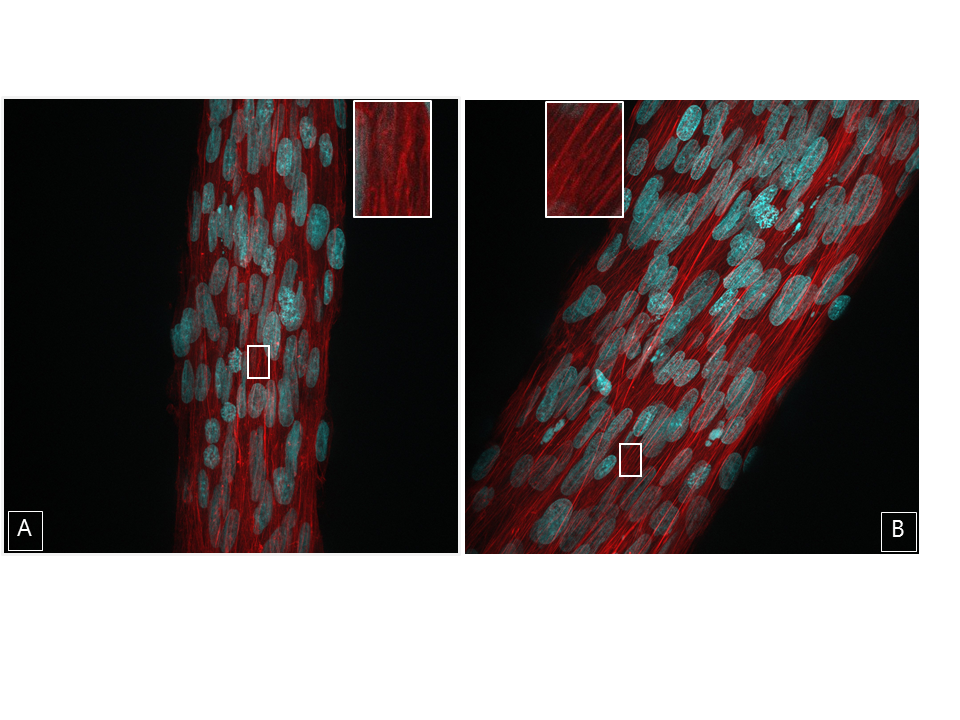


Fig.S2. Representative confocal images of human tendon constructs whole mount stained with phalloidin (F-actin, red) and counterstained with Hoechst (nuclei, blue). (A) Blebbistatin treated construct and (B) untreated control construct. Inset shows phalloidin stained actin stress fiber organization is disrupted by blebbistatin treatment.


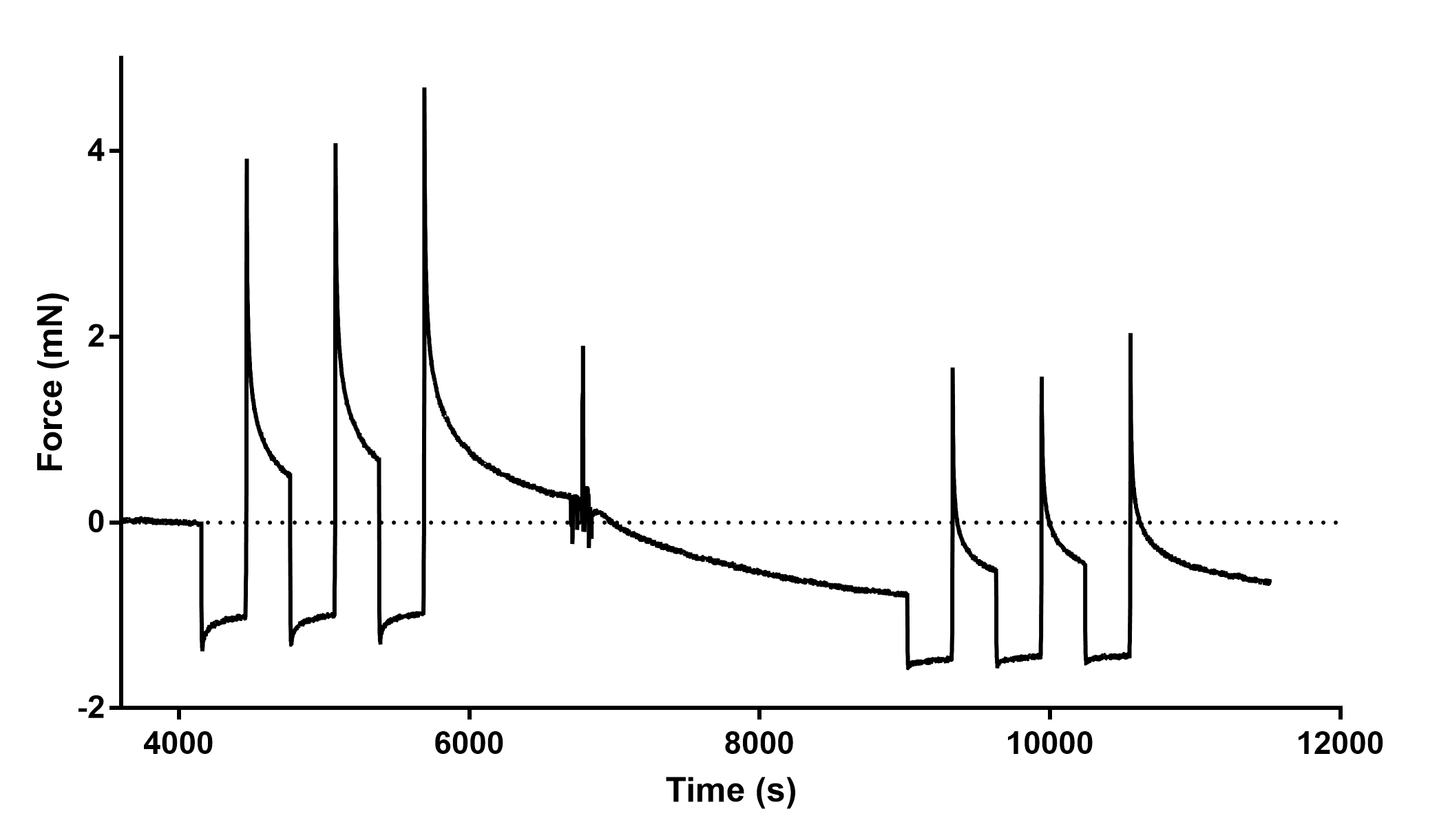

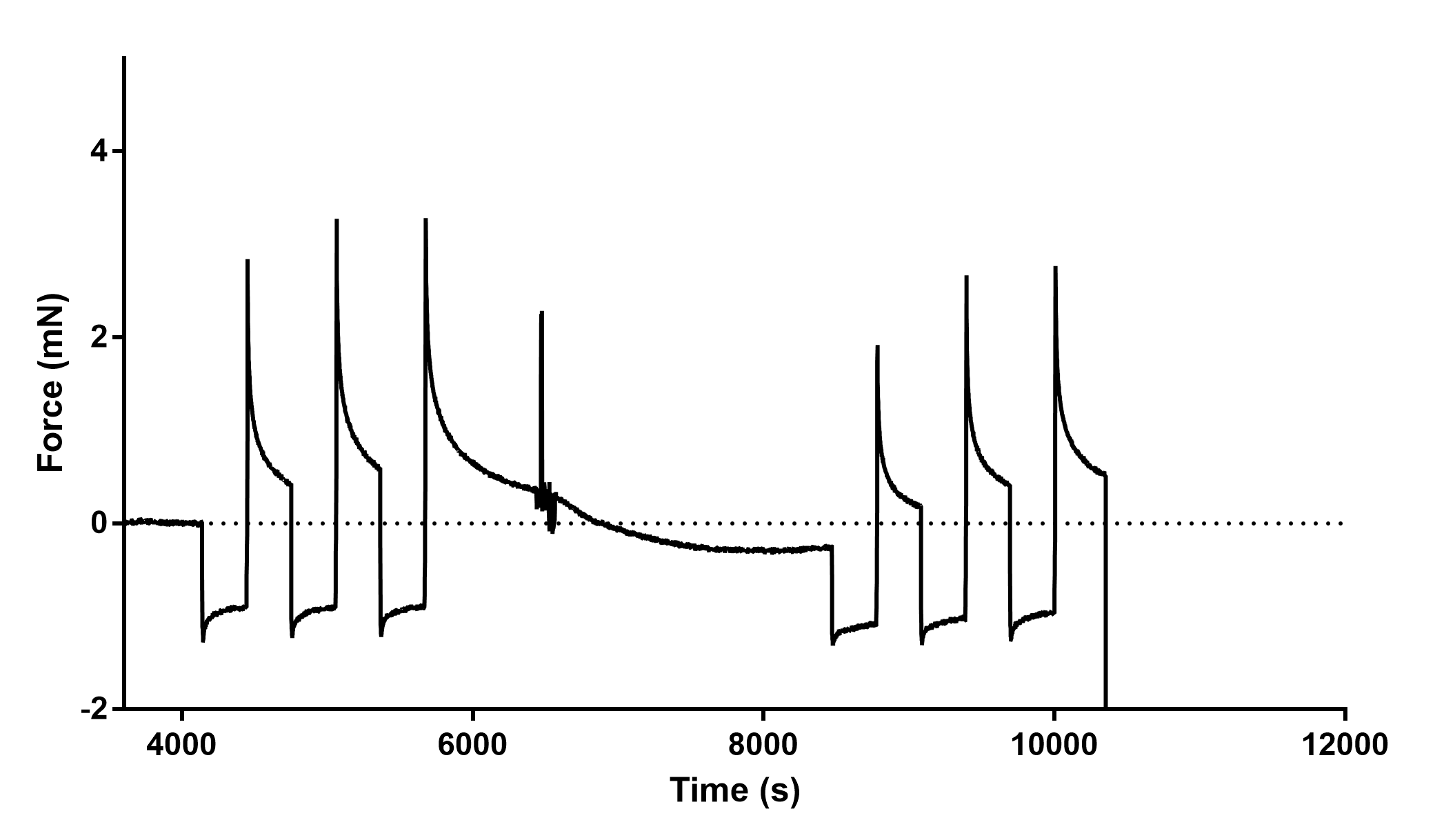


Fig. S3. Representative graphs of construct mechanical evaluation. One hour after mounting the construct to the well, a 3-cycle cyclic protocol was performed. Then the medium was replaced with the treatment medium followed by a 30 min incubation time. Finally three post-treatment cycles were performed. Top: Blebbistatin treatment. The force dropped as it can be seen in the graph indicating loss of tension due to the treatment. Bottom: CH ABC treatment.

# Supplementary Tables

Table S1. Table of absolute forces (mN) measured in the four different phases of the mechanical test (see Fig.1 in the main article). Median [inter quartile range].

| Treatment | Time | Unload (A-B) | Re-tension (B-C) | Reload (C-D) | Relaxation (D-A) |
| --- | --- | --- | --- | --- | --- |
| Blebbistatin | Pre (n=24) | -2.16 [-4.34;-1.75] | 0.43 [0.29;0.67] | 5.49 [4.05;8.19] | -3.67 [-4.70;-2.83] |
| Blebbistatin | Post (n=24) | -1.17 [-2.63;-0.89] | 0.09 [0.07;0.29] | 3.41 [2.49;6.10] | -2.36 [-3.48;-1.59] |
| CH ABC | Pre (n=23) | -2.41 [-3.78;-1.75] | 0.41 [0.31;1.03] | 6.82 [4.18;9.32] | -4.13 [-6.29;-2.56] |
| CH ABC | Post (n=23) | -2.02 [-3.27;-1.24] | 0.32 [0.25;0.63] | 4.47 [3.40;6.89] | -2.90 [-4.31;-2.10] |
| Control | Pre (n=10) | -4.37 [-6.92;-3.55] | 1.69 [1.03;3.16] | 8.93 [6.95;11.14] | -5.99 [-7.21;-4.66] |
| Control | Post (n=10) | -4.55 [-7.53;-3.53] | 1.56 [1.05;2.64] | 9.04 [7.54;12.03] | -6.06 [-6.85;-5.31] |

**References**

Bayer, M.L., Yeung, C.Y., Kadler, K.E., Qvortrup, K., Baar, K., Svensson, R.B., Magnusson, S.P., Krogsgaard, M., Koch, M., Kjaer, M., 2010. The initiation of embryonic-like collagen fibrillogenesis by adult human tendon fibroblasts when cultured under tension. Biomaterials 31, 4889-4897.

Hoemann, C.D., 2004. Molecular and biochemical assays of cartilage components. Methods Mol Med 101, 127-156.

Schindelin, J., Arganda-Carreras, I., Frise, E., Kaynig, V., Longair, M., Pietzsch, T., Preibisch, S., Rueden, C., Saalfeld, S., Schmid, B., Tinevez, J.Y., White, D.J., Hartenstein, V., Eliceiri, K., Tomancak, P., Cardona, A., 2012. Fiji: an open-source platform for biological-image analysis. Nat Methods 9, 676-682.
